# Biochemical Characterization of Two Thermostable Xylanolytic Enzymes Encoded by a Gene Cluster of *Caldicellulosiruptor owensensis*

**DOI:** 10.1371/journal.pone.0105264

**Published:** 2014-08-15

**Authors:** Shuofu Mi, Xiaojing Jia, Jinzhi Wang, Weibo Qiao, Xiaowei Peng, Yejun Han

**Affiliations:** 1 National Key Laboratory of Biochemical Engineering, Institute of Process Engineering, Chinese Academy of Sciences, Beijing, China; 2 Institute of Agro-food Science and Technology, Chinese Academy of Agricultural Sciences, Beijing, China; 3 Shenyang Agricultural University, Shenyang, China; Laurentian University, Canada

## Abstract

The xylanolytic extremely thermophilic bacterium *Caldicellulosiruptor owensensis* provides a promising platform for xylan utilization. In the present study, two novel xylanolytic enzymes, GH10 endo-β-1,4-xylanase (Coxyn A) and GH39 β-1,4-xylosidase (Coxyl A) encoded in one gene cluster of *C.owensensis* were heterogeneously expressed and biochemically characterized. The optimum temperature of the two xylanlytic enzymes was 75°C, and the respective optimum pH for Coxyn A and Coxyl A was 7.0 and 5.0. The difference of Coxyn A and Coxyl A in solution was existing as monomer and homodimer respectively, it was also observed in predicted secondary structure. Under optimum condition, the catalytic efficiency (*k*_cat_/*K*_m_) of Coxyn A was 366 mg ml^−1^ s^−1^ on beechwood xylan, and the catalytic efficiency (*k*_cat_/*K*_m_) of Coxyl A was 2253 mM^−1^ s^−1^ on *p*NP-β-D-xylopyranoside. Coxyn A degraded xylan to oligosaccharides, which were converted to monomer by Coxyl A. The two intracellular enzymes might be responsible for xylooligosaccharides utilization in *C.owensensis*, also provide a potential way for xylan degradation *in vitro*.

## Introduction

Xylan is the main hemicellulose component constituting about 20–30% of the biomass of dicotyl plants (hardwoods and herbaceous plants) [1]. Thus xylan is considered as an abundant renewable resource for biofuels and industrial chemicals production. The backbone chain of xylan is formed by β-1,4-linked D-xylosyl residues, and decorated with branched heteropolysaccharides [2]. Endo-β-1,4-xylanase, mostly present in the glycoside hydrolase (GH) families 10 and 11, hydrolyzes the endo-β-1,4-xylosidic linkages in a random fashion, while the hydrolysis is influenced by sidechain substituents [3]. The β-xylosidase, found in GH families 3, 30, 39, 43, 51, 52, and 54, catalyzes the conversion of xylobiose and other short chain xylooligosaccharides to D-xylose [3]. Hence endo-β-1,4-xylanase [EC 3.2.1.8] and β-1,4-xylosidase [EC 3.2.1.37] take primary responsibility for xylan backbone degradation. To date, an increasing number of xylanases and xylosidases have been isolated [3]–[8], while the knowledge of synergism of extreme xylanolytic enzymes for natural xylan degradation is still limited. Therefore, developing novel thermostable xylanase and xylosidase active at even higher temperature is desirable.

Application of extremely thermophilic microorganisms for biomass conversion is a way to break through the barriers of feedstock fluctuations and process-operating challenges [9]. Genus *Caldicellulosiruptor*, obligatory anaerobic, extreme thermophiles, produces heat-stable extracellular enzyme for biomass degradation. Several xylanases and xylosidases have already been characterized from *Caldicellulosiruptor* [10]–[14], while there are still many predicted xylanolytic enzymes encoded by *Caldicellulosiruptor* un-characterized.

The optimum culture condition for *Caldicellulosiruptor owensensis* OL is 75°C and pH 7.5. It grows on a wide variety of carbon sources including pentose, hexose, oligosaccharide, and polysaccharides [15]. The physiological characteristics of *C.owensensis* make it a promising candidate for pentose utilization. The genome sequence of *C.owensensis* has been reported, and the diversity of encoded glycoside hydrolases were analyzed thereafter [16]–[18].

In present study, two novel genes of endo-β-1,4-xylanase and β-1,4-xylosidase encoded by a gene cluster of *C.owensensis* were investigated. The two xylanolytic enzymes were heterogeneously expressed, purified and biochemically characterized. The catalytic model of endo-β-1,4-xylanase and β-1,4-xylosidase versus xylo-oligosaccharides were investigated, and the synergism of the two enzymes for xylan backbone hydrolysis were also studied.

## Materials and Methods

### Bacterial strains, plasmids, chemicals and growth conditions

*Caldicellulosiruptor owensensis* was purchased as free-dried culture from the German Collection of Microorganisms and Cell Cultures (DSMZ), DSM number is 13100. *Escherichia coli* Top10 and BL21(DE3) were respectively used for gene cloning and expression. The plasmid, pET-28b(+) (Novagen, USA), was used for recombinant plasmid construction and gene expression. Beechwood xylan and 4-nitrophenyl β-D-xylopyranoside (*p*NPX) were purchased from Sigma-Aldrich (St louis, MO, USA). All of the other chemicals were of analytical grade and purchased from Sinopharm Chemical Regent Co., Ltd. *C.owensensis* cells were resuspended and subcultured in anaerobic modified DSMZ medium 640 (cellobiose was replaced by xylose), and grown at 75°C with orbital shaking (75 rpm). *E.coli* Top10 and BL21(DE3) were cultured in aerobic Luria–Bertani (LB) broth at 37°C with orbital shaking (220 rpm).

### Genomic DNA isolation and recombinant vector construction

For preparation of genomic DNA from *C.owensensis*, cultured cells were harvested by centrifugation at 10,000×g for 1 min, then resuspended in 180 µl lysozyme buffer (20 mM Tris-HCl, pH 8.0, 2 mM Na_2_EDTA, 1.2% Triton, lysozyme 20 mg/ml), and incubated at 37°C for 30 min. This suspension was mixed with 5 µl Rnase A (100 mg/ml), shaking for 15 s, then incubated for 5 min at room temperature. Isolation of genomic DNA was carried out according to the instruction of TIANamp Bacteria DNA Kit (Tiangen Biotech, Beijing, China).

*Coxyn A* gene was amplified from *C.owensensis* genomic DNA with PCR primers MI03 (5′-GGAATTCCATATGAGTGAATATCAAGATAAAAC-3′) and MI04 (5′-CCGCTCGAGTTAAAAATTAACAATCCTGAAAAATG-3′). The PCR product was purified and digested by Nde I/Xho I, and ligated into pET28b(+) vector with same digestion. Similarly, the PCR primers for *Coxyl A* gene cloning were MI17 (5′-GCCGCGCGGCAGCATGAAAATAGAACTTTAC-3′) and MI18 (5′-GCGGCCGCAAGCGTTTACTCAGTTTTTATCG-3′), the PCR product was purified and treated by T4 DNA polymerase (TaKaRa Bio, Otsu, Japan) to produce cohesive ends, and then ligated with pET28b(+) vector with same sticky ends. The ligation mixture was transformed into *E.coli* TOP10 competent cells, clones were screened by using colony PCR, and positive clones were identified by sequencing.

### Protein expression and purification

To express *Coxyn A* and *Coxyl A*, the recombinant DNA plasmids were extract using TIANprep mini plasmid kit (Tiangen Biotech, Beijing, China), and transformed into *E.coli* BL21 (DE3) competent cells. The transformed strain was cultured in LB broth with 50 µg/ml kanamycin overnight at 37°C. The culture was diluted 100-fold in 500 ml fresh LB broth with 50 µg/ml kanamycin and grown to OD_600_ 0.6, isopropyl-β-D-thiogalactopyranoside (IPTG) (final concentration of 0.4 mM) was added to induce protein expression, and the culture was cultivated for another 6 hours at 37°C. To purify the protein, the culture was centrifuged at 6,000×g for 15 min and resuspended in 25 ml binding buffer (50 mM Tris-HCl, pH 7.5, 300 mM NaCl). The cells were broken by sonication, the mixture was then incubated at 65°C for 30 min, and centrifuged at 13,000×g for 15 min. The supernatant was passed five times through the Ni^2+^-NTA column to binding the histidine-tagged target protein. After washing three times to remove the non-specific protein, bound proteins was eluted from column with elution buffer (500 mM imidazole, 50 mM Tris-HCl, pH 7.5, 300 mM NaCl), and analyzed by 12% SDS-PAGE electrophoresis. To determine protein quaternary structure, gel filtration chromatography with Superdex 200 column was used. The column was pre-equilibrated with citrate buffer (50 mM sodium citrate, pH 6.0, 150 mM NaCl), and calibrated using carbonic anhydrase (30.0 kDa), Albumin (66.0 kDa), lactate dehydrogenase (140 kDa) catalase (232 kDa), ferritin (440 kDa) and thyroglobulin (669 kDa) as standards, the molecular masse (Mw) of proteins was estimated from a regression curve by plotting log of Mw standards against their elution volumes of the column. After gel filtration, fractions were analyzed by 12% SDS-PAGE electrophoresis, and the targeted proteins were collected and concentrated by ultrafiltration.

### Enzyme assay

The concentration of purified protein was determined by the Coomassie Brilliant Blue G250 binding method using bovine serum albumin solution to make standard curve [19]. To measure the xylanase activity of Coxyn A, 99 µl beechwood xylan (2.5 mg/ml) in citrate buffer (50 mM sodium citrate, pH 6.0, 150 mM NaCl) was incubated at 75°C for 5 min, then add 1 µl enzyme (24 µg/ml) into solution to react for 90 s. The reaction was terminated by heating for 10 min at 100°C (boiling). Then the reaction mixture was centrifuged at 13,000 rpm for 10 min. the released reducing sugar in supernatant was measured with PAHBAH (*p*-Hydroxy benzoic acid hydrazide) method [14], [20] and was monitored at the absorbance at 410 nm. One unit of enzyme activity was defined as the amount of enzyme capable of releasing 1 µmol of reducing sugar from beechwood xylan per minute. To measure the activity of Coxyl A, 99 µl *p*NP-β-D-xylopyranoside (*p*NPX) (0.5 mM) in citrate buffer (50 mM sodium citrate, pH 6.0, 150 mM NaCl) was incubated at 75°C for 5 min,1 µl enzyme (46 µg/ml) was added into solution to react for 2 min. The reaction was terminated by adding 200 µl 2 M Na_2_CO_3_ (pH 10) and monitored at the absorbance at 400 nM. One unit of β-Coxyl Activity was defined as the amount of enzyme capable of releasing 1 µmol *p*NP from the substrates per minute.

The optimum temperature for Coxyn A and Coxyl A was determined over the range of 40-100°C. To get the optimum pH of enzymes, different buffer pH 4.0–6.5 (citrate buffer, 50 mM, 150 mM NaCl), pH 7.0–8.5 (phosphate buffer, 50 mM, 150 mM NaCl) were prepared. At the optimum temperature, the specific activity of enzyme was checked at different pH. Thermal stability of enzyme was monitored based on optimum temperature and pH, the enzyme residual activity was determined using standard methods after incubating the enzyme at 70, 75 and 80°C without substrate for different time (0.5, 1, 2, 3, 4, 6 h). The relative activity of enzyme was calculated with the initial activity as 100%.

In citrate buffer (50 mM sodium citrate, 150 mM NaCl) and at 75°C,the effects of different chemicals on the Coxyn A and Coxyl A activity were measured with pH value of 7.0 and 5.0, respectively. The chemicals NaCl, KCl, CaCl_2_, FeCl_2_, NiCl_2_, CoCl_2_, MnCl_2_, CuCl_2_, and MgCl_2_ were added at concentrations of 1 mM and 5 mM individually [21]. Ethanol, n-butanol, and isopropanol were added individually to the reaction at concentration of 5% (v/v) and 10% (v/v). 0.1% or 0.5% of SDS and Tween-20 were added to the reaction respectively. Enzyme activity was measured by standard methods as describe above at optimum react conditions. The relative activity of enzyme was calculated with untreated control as 100%.

### Kinetic parameter assay

The activity of Coxyn A was measured in citrate buffer (pH 7.0, 75°C) containing 0.5 to 5 mg/ml beechwood xylan, and activity of Coxyl A was measured in citrate buffer (pH 5.0, 75°C) containing 0.05 to 2.4 mM *p*NPX by methods described above. Using software Graphpad Prism 6, the Michaelis-Menten constant (*K*_m_) and the maximum velocity (*V*_max_) were estimated with a nonlinear regression (curve fit) and Michaelis-Menten equation. The *k*_cat_ value was calculated as the ratio of *V*_max_ to the concentration of enzyme used in the reaction.

### Hydrolytic properties on xylooligosaccharides and xylan

To determinate the hydrolytic products, substrate of 0.6 mg/ml beechwood xylan or 4 mg/ml xylooligosaccharides were prepared. The substrate was mixed with 100 µg/ml Coxyn A, or 3 mg/ml Coxyl A, or 100 µg/ml Coxyn A and 3 mg/ml Coxyl A respectively. The reaction mixture in citrate buffer (50 mM sodium citrate, pH 6.0, 150 mM NaCl) was incubated at 75°C for 12 h (with beechwood xylan) or 3 h (with xylooligosaccharides). After incubation, hydrolytic products were analyzed by thin-layer chromatography (TLC) assay, reducing sugar assay and high-performance liquid chromatography (HPLC) assay. For TLC assay, aliquots were spotted onto silica gel plates at intervals, xylose (X1) and xylooligosaccharides (X2-X4) were used as standards. The plates were developed with 1-butanol: acetic acid: water (10: 5: 1, v/v/v) as a mobile phase. For visualization of hydrolytic products, TLC plates were sprayed with a staining solution of methanolic orcinol (0.05%, w/v) and sulfuric acid (5%, v/v), and then heated for 10 min at 75°C for color development. Reducing sugar assay was carried out by PAHBAH method described above. Before HPLC assay, diluted hydrolytic products were filtered through a 0.2 µm filter, isolation of xylose and xylooligosaccharides was performed with Hi-Plex Ca column (7.7×300 mm, Agilent Technology, USA) using LC-20AT pump (Shimadzu, Japan) with water at a flow rate of 0.6 ml/min at 85°C. Samples were injected with 10 µl volume, and monitored using RID10A refractive index detector (Shimadzu, Japan). The xylose content of hydrolytic products was measured by quantifying its peak area with D-xylose standard curve (R^2^ = 0.9999868).

### Phylogenetic tree and sequence alignment of Coxyn A and Coxyl A with other thermostable enzymes

Amino acids sequences of Coxyn A and Coxyl A were submitted to NCBI database Blastp (Basic Local Alignment Search Tool for Protein, version 2.2.29+) respectively, and the characterized thermostable proteins with high identity to Coxyn A or Coxyl A were selected to prepare phylogenetic trees. The MUSCLE (multiple sequence comparison by log-expectation) program was used for multiple sequences alignments, and MEGA 6 software was used to plot phylogenetic trees of alignments by the neighbor-joining method.

### Statistical analysis

Experiments were repeated three times, results were shown as mean ± SD, and student's t-test for comparisons were carried out by EXCEL T.TEST function. Values of P<0.01 were considered significant.

## Results and Discussion

### Gene cloning and bioinformatic analysis

**Figure 1:**
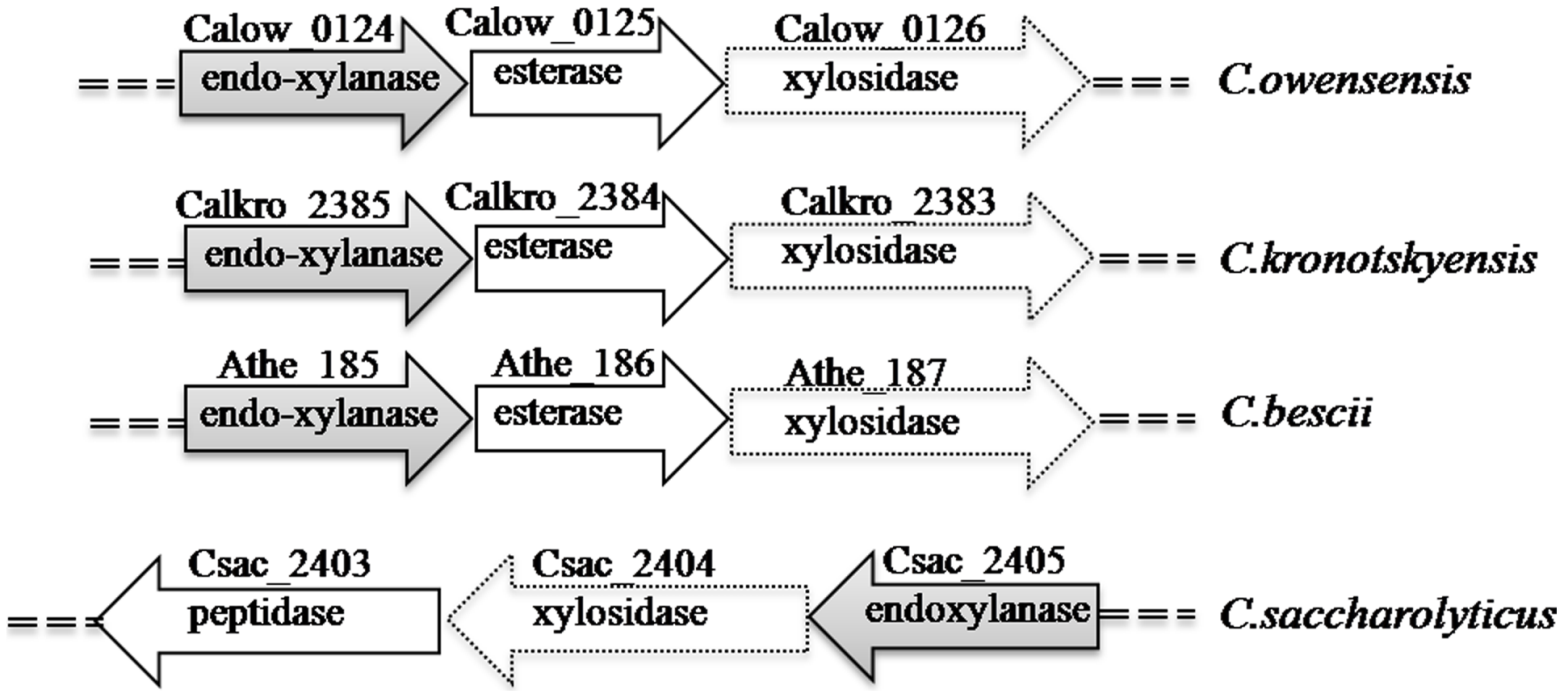
Gene cluster encoding xylanase, xylosidase, and esterase in genome of *C.owensensis*, *C.kronotskyensis*, *C.bescii*, and *C.saccharolyticus*.

The genes encoding glycoside hydrolases in *C.owensensis* genome were screened by using KEGG GENOME and Rapid annotation using subsystem technology (RAST). Five new predicted xylanases and two new xylosidases were identified in genome through amino acids similarity analysis. Among the genes, two of them Calow_0124 and Calow_0126 were identified in a gene cluster predicted for xylan degradation and metabolism. The genes Calow_0124 and Calow_0126 encoded a glycoside hydrolase (GH) 10 endo-β-1,4-xylanase of 337 amino acids and a GH39 β-1,4-xylosidase of 482 amino acids respectively. In the genes cluster of *C.owensensis*, a gene (Calow_0125) predicted as xylan esterase located between the two xylanolytic genes. Similar genes arrangement was observed in *C.kronotskyensis* and *C.bescii* (Fig. 1). In genome of *C.saccharolyticus*, corresponded gene cluster encodes a predicted peptidase (Csac_2403) except xylosidase (Csac_2405) and xylanase (Csac_2404). Based on amino acids analysis, all of the identity of Calkro_2385, Athe_185, and Csac_2405 with Calow_0124 was 81%. The amino acids identity of Calkro_2383, Athe_187, and Csac_2404 with Calow_0126 was 83–84%.

Both Coxyn A and Coxyl A have no signal peptide predicted by SignalP 4.1 server, suggesting the two enzymes might be intracellular enzymes. The secondary structures of the two enzymes were also predicted by GOR4 software. Results showed that Coxyn A contains 47.5% alpha helix (160 aa), 20.5% β-sheet (69 aa) and 32.0% random coil (108 aa); while Coxyl A has 29.9% alpha helix (144 aa), 22.6% β-sheet (109 aa) and 47.5% random coil (229 aa). That meant that nearly half secondary structure of Coxyn A was alpha helix, while most part of Coxyl A exists as random coil (Table 1). The difference of the two enzymes in secondary structure might contribute to the intermolecular interaction, as Coxyn A and Coxyl A exist as monomer and homodimer in buffer.

**Table 1:** Characterization of Coxyn A and Coxyl A.

Protein name	Genome locus tag a	Length (aa)	Mw (kDa)	Signal peptide b	GH family^c^	Predicted secondary structure^d^		
						Alpha helix	β-sheet	Random coil
Coxyn A	Calow_0124	337	40.2	No	GH10	160 aa	69 aa	108 aa
Coxyl A	Calow_0126	482	55.4	No	GH39	144 aa	109 aa	229 aa

a ^c^: Predicted in The National Center for Biotechnology Information (NCBI).

b : Predicted by SignalP 4.1 server.

d : Predicted by GOR4 software.

### Analysis of homologous sequence and active site prediction

**Figure 2:**
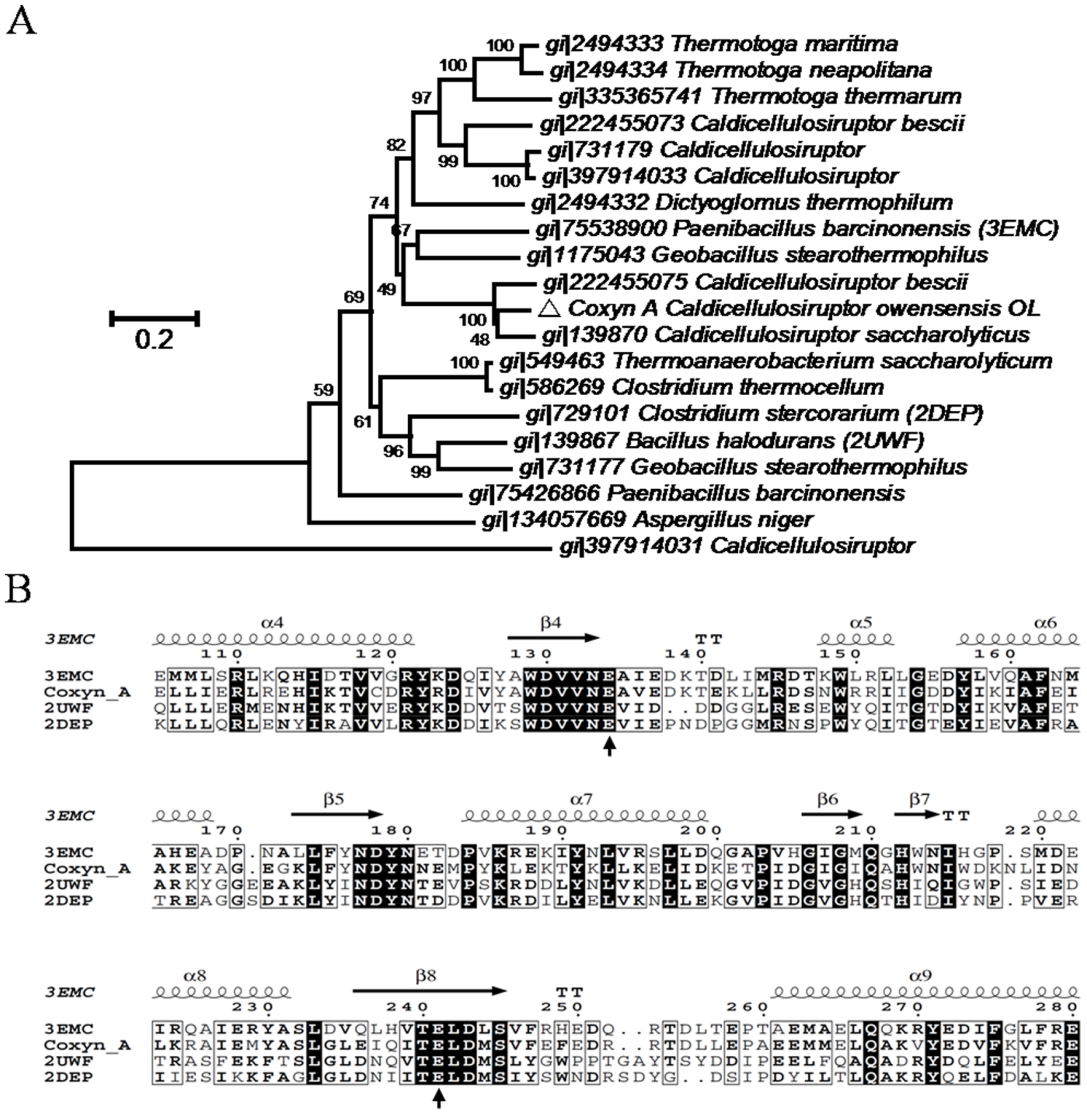
The phylogenetic trees of Coxyn A with similar enzymes. **A** The relationship of Coxyn A (Δ) from *C.owensensis* with other similar GH10 xylanases retrieved from NCBI database. **B** Sequence alignment of Coxyn A with those structure elucidated xylanses. The phylogenetic trees were constructed by MUSLE program and MEGA 6 Neighbor-joining analysis. Bootstrap values obtained with 1000 resamplings were indicated as percentages at all branches, and the scale bars represent 0.05 substitutions per amino acid position. Numbers followed by the names of the strains were accession numbers of NCBI.

**Figure 3:**
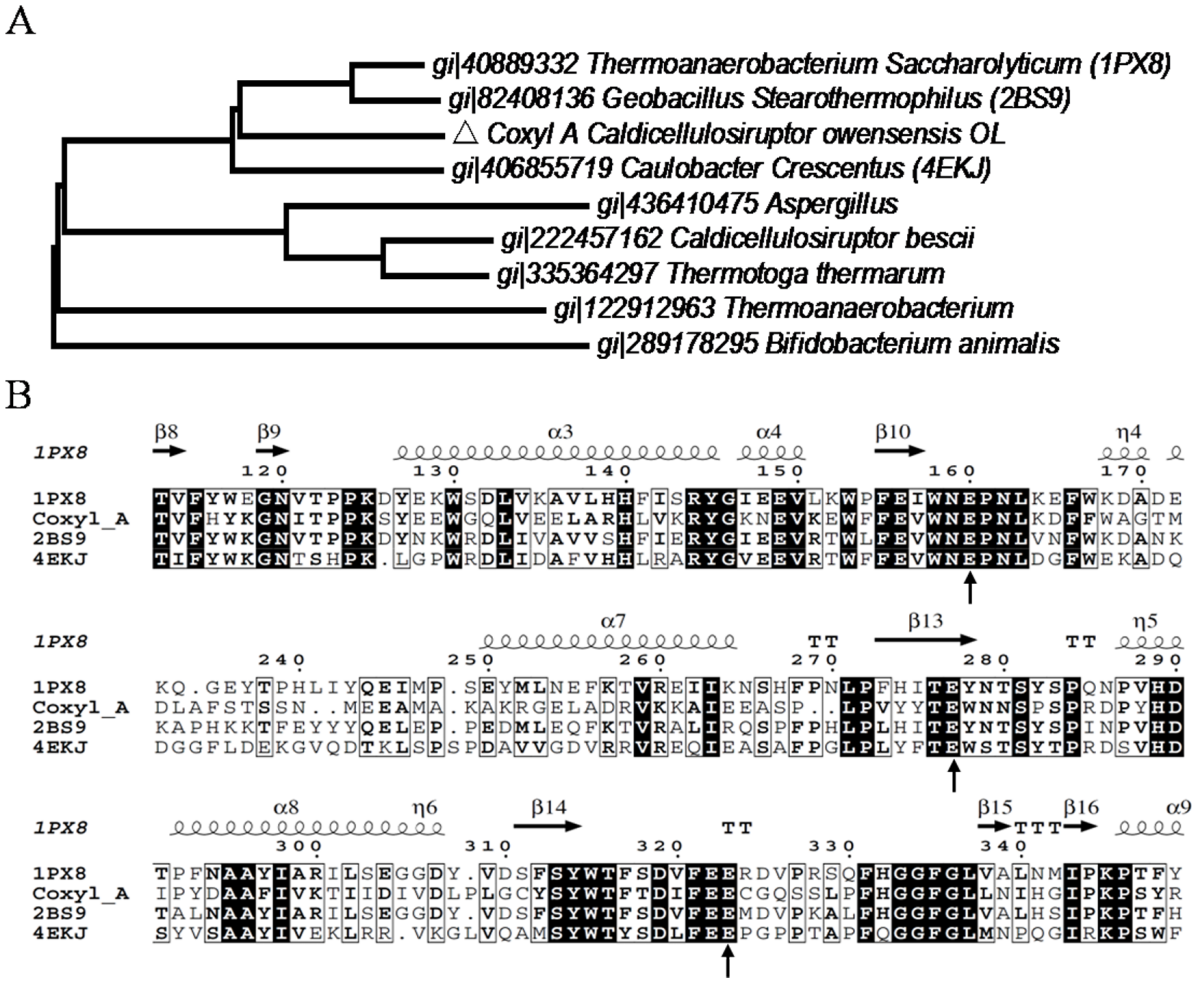
The phylogenetic trees of Coxyl A with similar enzymes. **A** The relationship of Coxyl A (Δ) from *C. owensensis* with other similar GH39 xylosidases retrieved from NCBI database. **B** Sequence alignment of Coxyn A with those structure elucidated xylanses. The phylogenetic trees were constructed by MUSLE program and MEGA 6 Neighbor-joining analysis. Bootstrap values obtained with 1000 resamplings were indicated as percentages at all branches, and the scale bars represent 0.05 substitutions per amino acid position. Numbers followed by the names of the strains were accession numbers of NCBI.

The homologous sequence of Coxyn A and Coxyl A with other xylanases and xylosidases was analyzed through phylogenetic trees, which were constructed based on amino acids sequences. The homology of Coxyn A was analyzed with the most similar characterized GH10 xylanase (amino acids sequence identity was equal or greater than 51%)(Fig. 2A), Coxyl A was compared with other GH39 xylosidase (amino acids sequence identity was equal or greater than 35%) (Fig. 3A). The phylogenetic tree of GH10 xylanase showed that Coxyn A from *C.owensensis* OL was the closest to GH10 xylanase from *C.saccharolyticus* and *C.bescii*, and they were separated from all other similar GH10 xylanases, which were from *Paenibacillus*, *Geobacillus* and *Bacillus* etc. In the listed GH10 enzymes, three mostly closed xylanase from PDB had been aligned with Coxyn A by ClustalW software. With protein 3EWC (PDB ID) as template, the result of ESPrit 3.0 software showed that Coxyn A had tipical (β/α)_8_ elements of GH10 [22]. The conserved catalytic residues (the acid/base Glu in β4 and the nucleophile Glu in β8) were also showed [23]. It suggested that Coxyn A probably had similar structure and catalytic mechanism with most GH10 enzymes (Fig. 2B).

Similar result appeared in the phylogenetic tree of GH39 xylosidases. These results illustrated that the amino acids sequence of Coxyn A and Coxyl A are special to other enzymes, and suggesting the novelty of the two xylanolytic enzymes encoded by gene cluster. Coxyl A was also aligned with mostly closed xylosidases from PDB by ClustalW, and secondary structure of Coxyl A was depicted by ESPrit 3.0 software by protein 1PX8 (PDB ID). The results showed that Coxyl A also had tipical (β/α)_8_ of GH39 and conserved catalytic residues (acid/base Glu^160^ and nucleophile Glu^277^) and substrate recognition residue (Glu^323^) [24]. Similar structure and catalytic mechanism of GH39 enzyme may be used for Coxyl A analysis (Fig. 3B).

### Expression and purification of Coxyn A and Coxyl A

**Figure 4:**
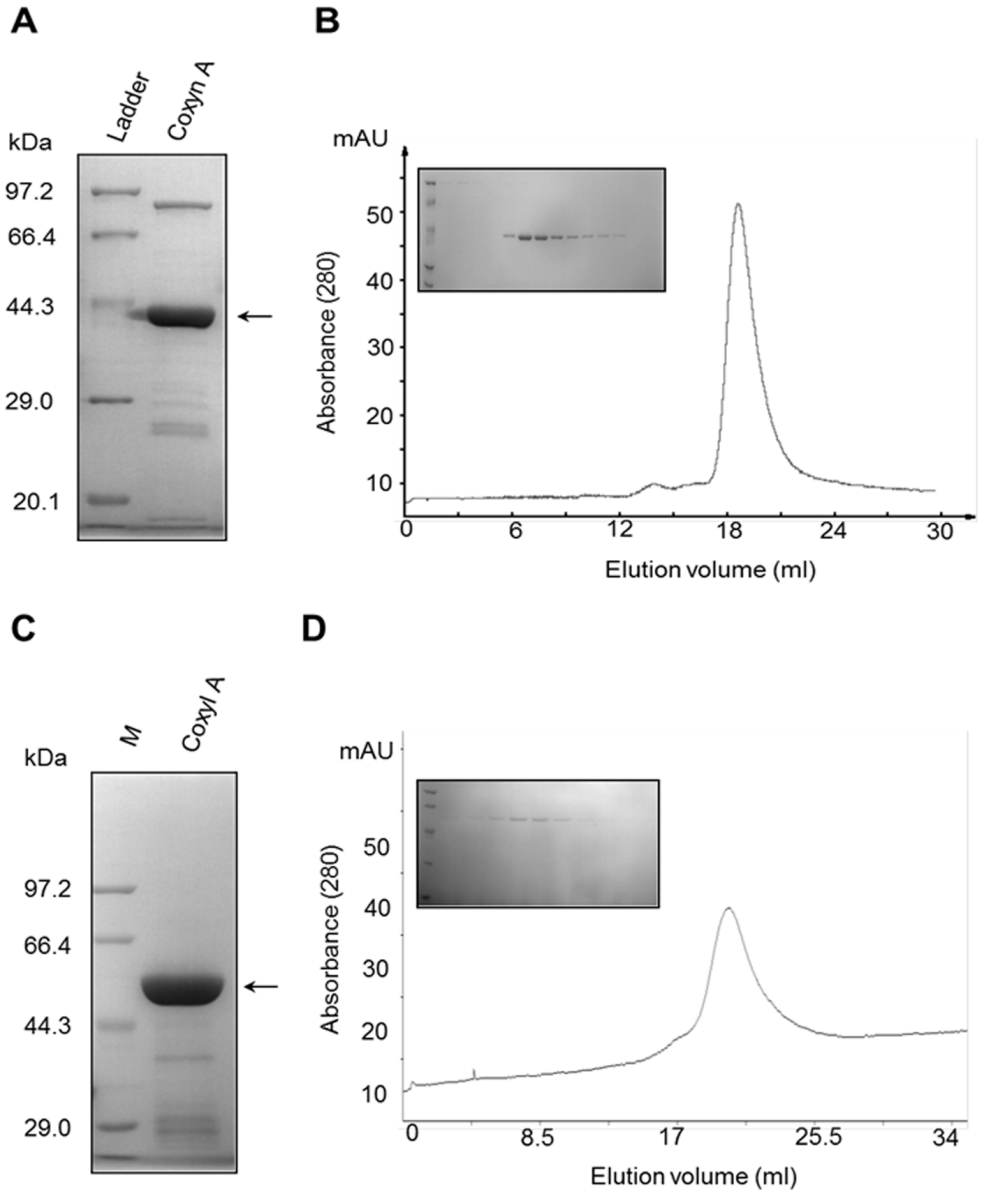
Expression, purification, and quarternary structure of Coxyn A and Coxyl A. **A** SDS-PAGE of Coxyn A purified by affinity chromatography on Ni^2+^-NTA-Sepharose 4B. **B** Coxyn A was a monomer by Gel filtration chromatography on a Superdex-200 column (1.1×26.0 cm) and SDS-PAGE electrophoresis. **C** SDS-PAGE of Coxyl A purified by affinity chromatography on Ni^2+^-NTA-Sepharose 4B. **D** Coxyn A was a homodimer by Gel filtration chromatography on a Superdex-200 column (1.0×40.0 cm) and SDS-PAGE electrophoresis.

The recombinant Coxyn A and Coxyl A were expressed in *E. coli* BL21 (DE3), then purified with affinity chromatography (Ni2+-NTA resins) and gel filtration chromatography. The SDS-PAGE (12%) analysis of Coxyn A after affinity chromatography was shown in Fig. 4A. The contaminant bands were removed by gel filtration, purified Coxyn A existed as monomer (40 kDa) (Fig. 4B). Similarly, after affinity chromatography, Mw of Coxyl A was about 55 kDa analyzed by 12% SDS-PAGE (Fig. 4C), the enzyme existed as homodimer through gel filtration analysis (Fig. 4D). Most reported β-xylosidase existed as monomer with a exception of *Bacillus pumilus* homodimeric β-xylosidase [25].

### Biochemical characterization of Coxyn A and Coxyl A

**Figure 5:**
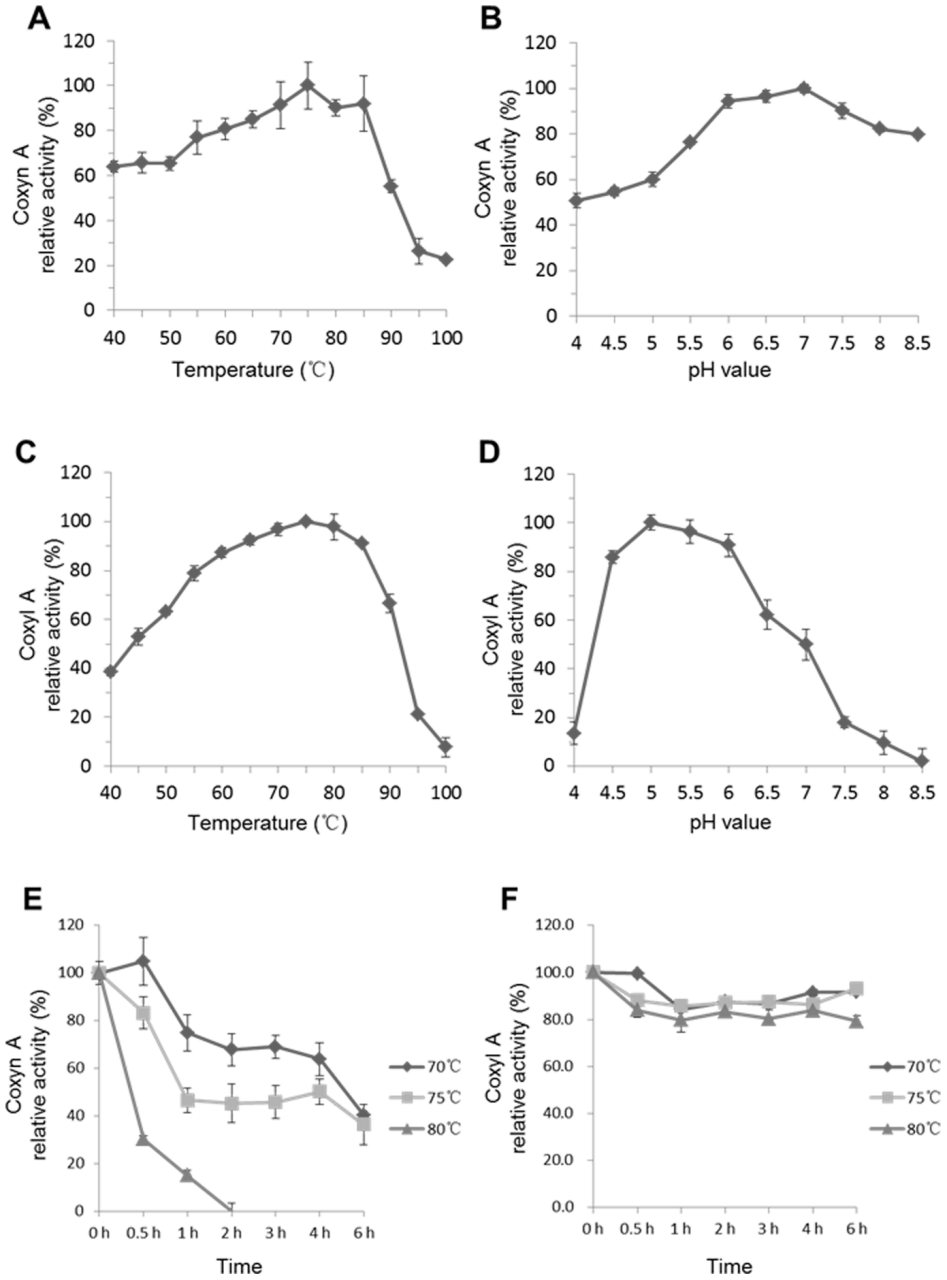
Temperature and pH profile of Coxyn A and Coxyl A. **A** Effect of temperature on Coxyn A activity. **B** Effect of pH on Coxyn A activity. **C** Effect of temperature on Coxyl A activity. **D** Effect of pH on Coxyl A activity. **E** Effect of temperature on Coxyn A stability. **F** Effect of temperature on Coxyl A stability.

In temperature profile analysis, Coxyn A showed highest activity at 75°C and retained more than 80% activity at temperature range of 60–85°C (Fig. 5A). Coxyn A retained 50% activity at pH range from 4.0 to 8.5 and optimum pH was 7.0 (Fig. 5B). The temperature feature of Coxyl A activity was similar to Coxyn A, and the optimum temperature was 75°C (Fig. 5C). In contrast, Coxyl A was active at acidic condition (pH range of 4.5 to 6.0), with an optimum pH of 5.0 (Fig. 5D). The genes encoding Coxyn A and Coxyl A were located in same gene cluster, while the optimum pH of the two enzymes is different. As the interesting results, it might be speculated that *C.owensensis* encoded seven xylanses and xylsidase adapting to different condition, while the speculation need to be tested.

For determination of thermostability, Coxyn A and Coxyl A were pre-incubated at different temperature (70, 75, and 80°C) for 0.5–6 h in citrate buffer with optimum pH. The results showed that Coxyn A was not very stable at optimum temperature, the residual activity retained only about 40% at 70°C and 75°C after 2 h's incubation. After incubating for 2 h at 80°C, no activity of Coxyn A was observed (Fig. 5E). However, Coxyl A was stable after incubating for 6 h at 70°C, 75°C and 80°C (Fig. 5F). It might be the homodimeric form of Coxyl A that stabilizes its structure, and reduced the loss of enzyme activity during the incubation. It should be noted that both Coxyn A and Coxyl A were stable at room temperature for several days. The thermostability of Coxyl A from *C.owensensis* makes it useful in biotechnological processing of biomass.

To optimize the condition for catalytic activity, the effects of metal ions and chemicals on enzyme activities were investigated (Table 2). For Coxyn A, its activity was influenced slightly by smaller atomic monovalent and divalent cations and their chelating agent, i.e. Na^+^, Ca^2+^, and Mg^2+^, while K^+^ was a exception. Heavier metal cations had different influence, Co^2+^ and Cu^2+^ had significant positive influence on Coxyn A activity, especially at 5 mM concentration. Ni^2+^ and Mn^2+^ had more negative effect on enzyme activity at 1 mM concentration than at 5 mM, and the negative influence of K^+^ and Fe^2+^ grew with their concentration increased. In organic reagents, ethanol, n-butanol and isopropanol, but not glycerol, had negative effect on Coxyn A activity. Except for SDS, several surfanctants had no negative effect on Coxyn A activity. Different from Coxyn A, Coxyl A activity was enhanced by adding surfanctants such as tween-20 and Glycerol. While SDS, ethanol, n-butanol and isopropanol showed negative effects on Coxyl A activity. Furthermore, metal ions had no or slightly effect on Coxyl A activity except for the negative influence of Fe^2+^.

**Table 2:** Effect of chemical reagents on the activity of Coxyn A and Coxyl A.

Chemicals	Relative activity (%)^1^			
	Coxyn A		Coxyl A	
	1 mM	5 mM	1 mM	5 mM
Control	100.0±1.1	100.0±1.7	100.0±0.5	100.0±0.8
NaCl	112.8±6.3	89.8±5.5	102.1±0.4	98.0±1.6
KCl	100.1±2.3	32.6±2.9	102.4±0.2	97.3±1.2
CaCl_2_	100.2±1.9	102.5±1.7	102.4±2.7	96.1±2.4
FeCl_2_	77.1±7.7	33.0±2.3	88.5±0.5	48.7±1.2
NiCl_2_	33.5±3.6	82.3±18.4	96.2±0.7	69.9±3.0
CoCl_2_	102.0±5.6	151.1±6.1	98.2±2.2	81.3±1.1
MnCl_2_	44.6±3.2	78.8±7.0	98.4±2.9	86.2±2.6
CuCl_2_	113.3±5.8	168.9±17.4	85.7±0.7	61.5±0.7
MgCl_2_	88.9±1.1	117.3±1.1	102.9±2.2	95.0±0.4
	5%	10%	5%	10%
Glycerol	173.9±2.2	185.6±4.2	63.5±0.4	41.1±1.3
Ethanol	34.3±1.1	0.3±4.0	101.1±1.8	94.9±1.5
n-Butanol	0	0	104.3±8.3	57.4±4.5
Isopropanol	45.7±7.4	0	114.0±3.6	107.5±6.3
	0.1%	0.5%	0.1%	0.5%
SDS	5.6±5.9	0	8.4±1.9	11.5±0.6
Tween-20	102.8±9.0	111.3±13.5	151.2±0.5	123.3±2.4

1 Data are means ± SD (n = 3) relative to the control samples.

### Enzyme kinetic parameters of Coxyn A and Coxyl A

The kinetic parameters for Coxyn A were determined under the optimum condition of 75°C and pH7.0. As shown in Table 3, with beechwood xylan as substrate, the values for *K*_m_, *V*_max_ and *k*_cat_ of Coxyn A were 0.62±0.33 mg/ml, 341.4±47.1 µmol mg^−1^ min^−1^ and 227±31.3 s^−1^, respectively. Compared with the similar xylanases characterized and listed in Fig. 5, Coxyn A was a xylanase with high efficiency at high temperature and neutral environment. The kinetic parameters of Coxyl A were shown in Table 4, and the respective values for *K*_m_, *V*_max_ and *k*_cat_ were 1.6 mM, 3930 µmol mg^−1^ min^−1^ and 3628 s^−1^ with *p*NPX as substrate. Compared to characterized xylosidase listed in Fig. 3, *K*_m_ of Coxyl A is lower and the *k*_cat_ of Coxyl A was the highest.

**Table 3:** Kinetic parameters of Coxyn A compared with other GH10 xylanases.

Resource	Substrate	*K*_m_ (mg ml^−1^)	*V*_max_ (µmol mg^−1^ min^−1^)	Kcat (s^−1^)	Optimum temperature(°C)	Optimum pH	Reference
*Caldicellulosiruptor owensensis*	BWX1	0.62±0.33	341.4±47.1	227±31.3	75	7.0	This work
*Caldicellulosiruptor saccharolyticus*	OSX	ND	2.7^a^	ND	70	5.5–6.0	[31]
*Aspergillus niger*	BWX1	2.8	127	76	55	5.5	[32]
*Clostridium stercorarium*	OSX	3.7	4500	ND	80	6.0	[33]
*Thermotoga neapolitana*	OSX	ND	111.3^a^	ND	102	5.5	[34]
*Thermotoga thermarum*	BWX1	1.8	769	520	80	6.0	[35]
*Thermoanaerobacterium saccharolyticum*	OSX	ND	227.4^a^	ND	70	5.5	[36]
*Caldicellulosiruptor sp. F32*	xylan	8.3	942.1±23.98^a^	ND	75	6.6	[13]
*Caldicellulosiruptor sp. F32*	xylan	10.7	103.6±2.5^a^	ND	75	6.6	[13]
*Caldicellulosiruptor bescii*	BWX2	1.3±0.5	ND	123.2±6.6	85	6.0	[37]
*Caldicellulosiruptor bescii*	OSX	3.5±1.1	ND	102.4±7.4	85	6.0	[37]
*Caldicellulosiruptor bescii*	BWX2	4.0±0.7	ND	198.1±7.6	80	6.5	[37]
*Caldicellulosiruptor bescii*	OSX	13.3±2.0	ND	400.0±20.9	80	6.5	[37]

a specific activity; BWX1: beechwood xylan; BWX2: birchwood xylan; OSX: oat splet xylan.

**Table 4:** Kinetic parameters of Coxyl A compared with other xylosidases^a^.

Organism	*K*_m_ (mM)	*V*_max_ (µmol mg^−1^ min^−1^)	*k*cat (s^−1^)	Optimum temperature(°C)	Optimum pH	Reference
*Caldicellulosiruptor owensensis*	1.6±0.1	3930±132	3628±122	75	5.0	This work
*Caldicellulosiruptor bescii*	8.2±0.2	ND	620.8±8.0	90	6.0	[14]
*Caulobacter crescentus*	9.3±0.45	402±19	ND	55	6.0	[38]
*Thermotoga thermarum*	0.27	223.2	316.8	95	6.0	[39]
*Bifidobacterium animalis subsp. lactis BB-12*	15.6±4.2	166	60.6±10.8	50	5.5	[40]
*Thermoanaerobacterium saccharolyticum*	28	189292	276	65	6.0	[41]
*Aspergillus sp. BCC125*	1.7	211.5	338	60	4.0–5.0	[28]
*Aspergillus phoenicis*	2.36±0.54	ND	920.75±40.45	75	4.0–4.5	[42]
^a^Substrate: *p*NPX						

### Hydrolytic property analysis

**Figure 6:**
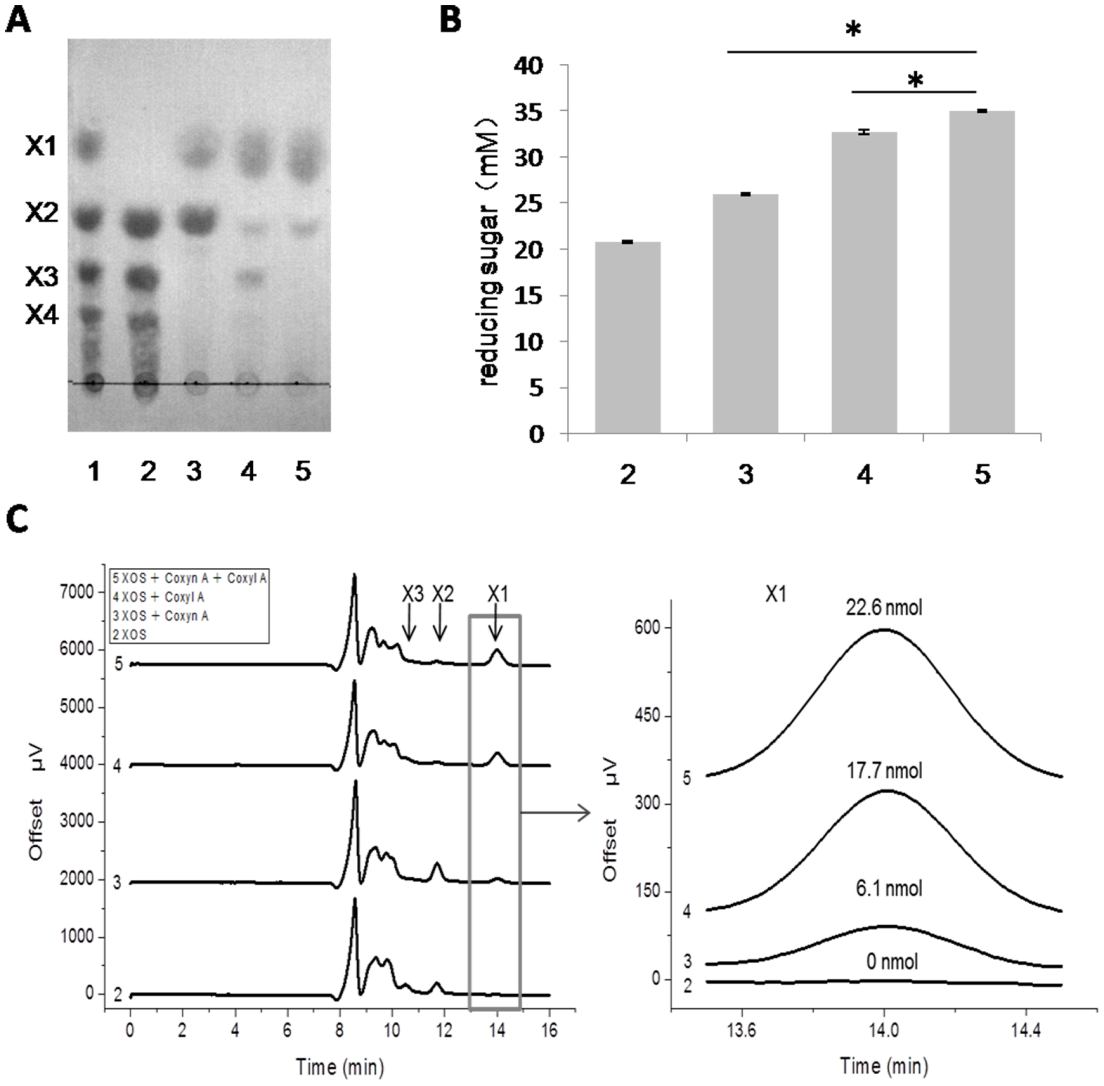
Hydrolytic mode of xylooligosaccharides by Coxyn A and Coxyl A. **A** Thin-layer chromatography (TLC) assay. lane 1: standards of xylose and xylooligosaccharides (XOS) (X1: xylose, X2: xylobiose, X3: xylotriose, X4: xylotetrose); lane 2: 4 mg/ml XOS; lane 3: 4 mg/ml XOS was incubated with 100 µg/ml Coxyn A; lane 4: 4 mg/ml XOS was incubated With 3 mg/ml co xylosidase A; lane 5: 4 mg/ml XOS was incubated With 100 µg/ml Coxyn A and 3 mg/ml Coxyl A. The reaction was conducted in citrate buffer (pH 6.0), 75°C for 3 h. **B** Reducing sugar assay of hydrolysis. *P<0.01 (n = 3), the number of sample means the same sample loaded on the line in (A). **C** High Performance Liquid Chromatography assay of products.

To determine the hydrolytic mode of Coxyn A and Coxyl A, xylooligasaccharides and xylan were applied as substrates. When the xylooligosaccharides were hydrolyzed with Coxyn A, the final products were xylose and xylobiose. As a contrast, the main product was xylose when xylooligosaccharides was hydrolyzed with Coxyl A. The results indicated that Coxyl A was active on oligosaccharides with DP≥2 (Fig. 6A). The products of xylooligosaccharides hydrolysis were also analyzed by HPLC assay. The concentrations of xylose of the control, Coxyn A, Coxyl A, and Coxyn A&Coxyl A were 0, 6.1, 17.7, 22.6 nmol, respectively (Fig. 6C). This result suggested that the combination of Coxyn A and Coxyl A could hydrolyze xylooligosaccharides into xylose more efficiently than they acted alone. Similar results were detected in reducing sugar assay, the final reducing sugars for the control, Coxyn, Coxyl A, and Coxyn&Coxyl A were 20.8±0.2, 26.0±0.1, 32.7±0.3, 35.1±0.1 mM. Significant difference at P<0.01 was observed in statistical analysis between Coxyn and Coxyn&Coxyl A, Coxyl A and Coxyn&Coxyl A (Fig. 6B).

**Figure 7:**
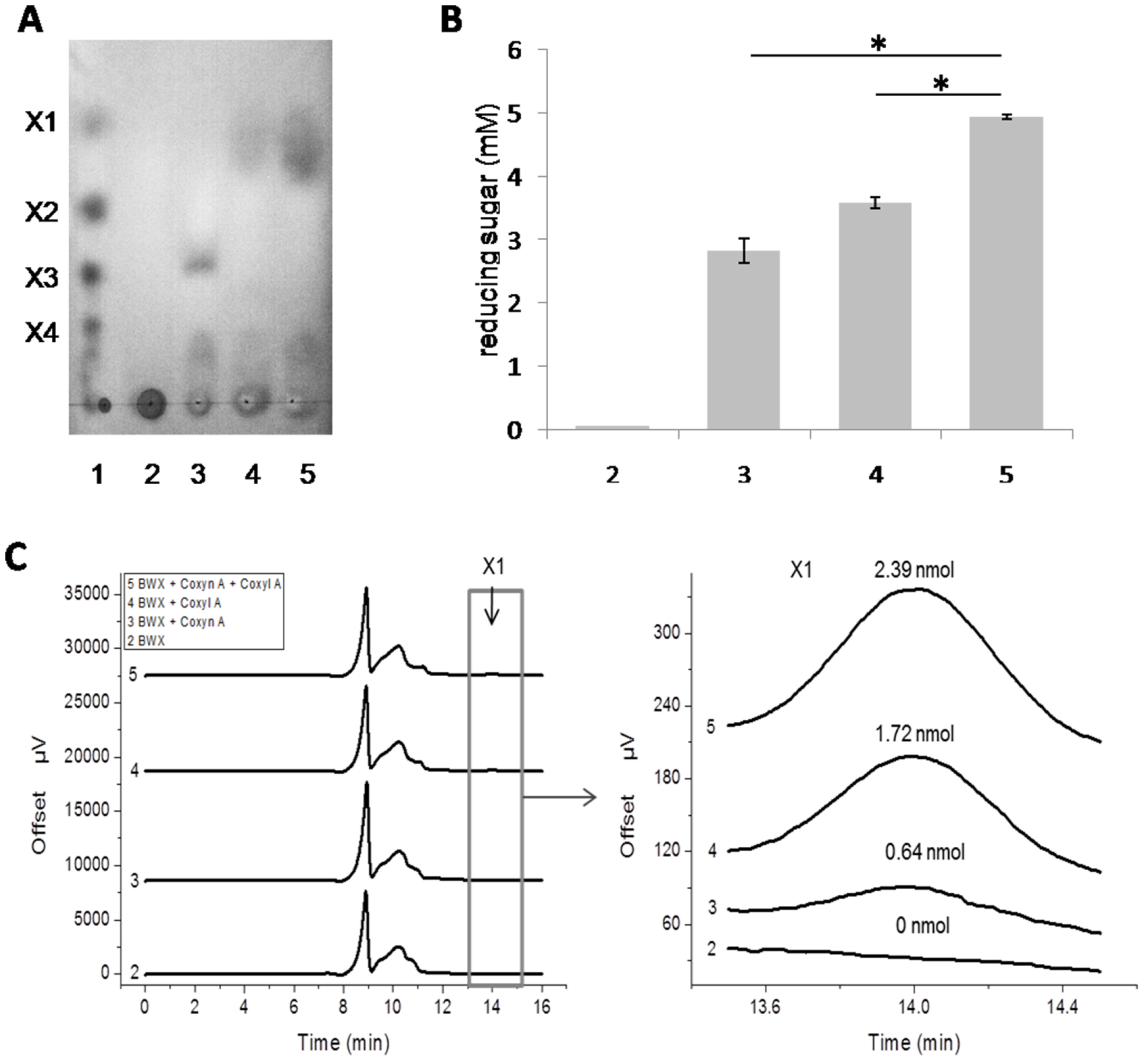
Hydrolytic mode of beechwood xylan by Coxyn A and Coxyl A. **A** TLC assay of beechwood xylan hydrolyzed with Coxyn A and Coxyl A. lane 1: standards of xylooligosaccharides (X1-X4); lane 2: 0.6 mg/ml beechwood xylan (BWX); lane 3: 0.6 mg/ml BWX was incubated With 100 µg/ml Coxyn A; lane 4: 0.6 mg/ml BWX was incubated With 3 mg/ml Coxyl A; lane 5: 0.6 mg/ml BWX was incubated With 100 µg/ml Coxyn A and 3 mg/ml Coxyl A. All above were in citrate buffer (pH 6.0), 75°C for 12 h. **B** Concentration of reducing sugar in the hydrolysis. The number of sample means the same sample loaded on the line in (A), *P<0.01 (n = 3). **C** HPLC assay of BWX hydrolysis.

Similar hydrolysis experiments were conducted using beechwood xylan as substrate. In hydrolytic products analysis through TLC assay, xylotriose and even longer xylooligosaccharides decorated with side chains were observed, while no xylobiose and xylose were found in the mixture. With Coxyl A was incubated with xylan, a small quantity of xylose was liberated, which might come from the degradation of small amount of xylooligosaccharides existing in the substrate. With combination of Coxyn A and Coxyl A, xylose and longer chain xylooligosaccharides exist, while no xylobiose or xylotriose were observed (Fig. 7A). The corresponded reducing sugar in the mixture was shown in Fig. 7B, the final amount of reducing sugar for the control, Coxyn, Coxyl A, and Coxyn&Coxyl A were 0.06±0.01, 2.8±0.2, 3.6±0.1, 4.9±0.03 mM. In statistical analysis, significant difference at P<0.01 was observed between Coxyn and Coxyn&Coxyl A, Coxyl A and Coxyn&Coxyl A. The products of beechwood xylan (diluted 10-fold) hydrolysis with different enzyme were also analyzed by HPLC assay. When Coxyn A was applied in the mixture, very small amount of xylose (0.6 nmol) was observed, which was under the detection limit of TLC. The amounts of xylose from reaction of control, Coxyn A, Coxyl A, and Coxyn A & Coxyl A were 0, 0.6, 1.7 and 2.4 nmol, respectively (Fig. 7C). To date, a increasing number of xylanases and xylosidases were characterized from bacteria to fungi [5], [7], [26]–[28], while two enzymes encoded by one gene cluster were rarely reported. It was reported that a synergy of two xylose-tolerant GH43 bifunctional β-xylosidase/α-arabinosidase and one GH11 xylanase from *Humicola insolens* acted in the degradation of xylan [29], other researchers even reconstituted a thermostable mixture of xylan-degrading enzymes from *C.bescii* for deconstruction of xylan [30]. In our experiment, the genes of GH10 Coxyn A and GH39 Coxyl A were found in one gene cluster, and the properties of which suggested a synergism for degradation of xylan.

## Conclusion

The genes for two novel xylanolytic enzymes GH10 endo-β-1, 4-xylanase and GH39 β-1, 4-xylosidase located in one gene cluster were found in genome of *C.owensensis*. Synergism of the two novel xylanolytic enzymes could efficiently convert the backbone of natural xylan to xylose at 75°C. The two xylanolytic enzymes are predicted to be intracellular enzymes and responsible for degrading polysaccharides or oligosaccharides to xylose. The high efficiency and excellent thermostability of Coxyn A and Coxyl A provide a potential way for xylan degradation *in vitro*.
